# Assessment of factors influencing retention in the Philippine National Rural Physician Deployment Program

**DOI:** 10.1186/1472-6963-12-411

**Published:** 2012-11-20

**Authors:** Juan Alfonso Leonardia, Helen Prytherch, Kenneth Ronquillo, Rodel G Nodora, Andreas Ruppel

**Affiliations:** 1 Institute of Public Health, University of Heidelberg, Heidelberg, Germany; 2 Present address: Deutsche Gesellschaft für Internationale Zusammenarbeit (GIZ), Makati, Philippines; 3 Department of Health, Health Human Resource Development Bureau, Manila, Philippines; 4 Human Resource Development, World Health Organization, Western Pacific Region, Manila, Philippines

**Keywords:** Developing countries, Health personnel, Retention, Job satisfaction, Rural health

## Abstract

**Background:**

The ‘Doctors to the Barrios’ (DTTB) Program was launched in 1993 in response to the shortage of doctors in remote communities in the Philippines. While the Program has attracted physicians to work in such areas for the prescribed 2-year period, ongoing monitoring shows that very few chose to remain there for longer and be absorbed by their Local Government Unit (LGU). This assessment was carried out to explore the reasons for the low retention rates and to propose possible strategies to reverse the trend.

**Methods:**

A mixed methods approach was used comprising a self-administered questionnaire for members of the current cohort of DTTBs, and oral interviews with former DTTBs.

**Results:**

Among former DTTBs, the wish to serve rural populations was the most widely cited motivation. By comparison, among the current cohort of DTTBs, more than half joined the Program due to return of service obligations; a quarter to help rural populations, and some out of an interest in public health. Those who joined the Program to return service experienced significantly less satisfaction, whilst those who joined out of an interest in public health were significantly more satisfied with their rural work. Those who graduated from medical schools in the National Capital Region were significantly more critical about their compensation and perceived there to be fewer options for leisure in rural areas. With regard to the factors impeding retention, lack of support from the LGU was most frequently mentioned, followed by concerns about changes in compensation upon absorption by the LGU, family issues and career advancement.

**Conclusions:**

Through improved collaboration with the Department of Health, LGUs need to strengthen the support provided to DTTBs. Priority could be given to those acting out of a desire to help rural populations or having an interest in public health, and those who have trained outside of the National Capital Region. Whether physicians should be able to use the Program to fulfil return service obligations should be critically assessed.

## Background

The problem of attracting, recruiting and retaining skilled health workers in rural areas has risen high on the agenda of policy-makers. It is a global problem, the effects of which are most pronounced in countries where staffing deficits are severe and where rural areas are particularly inaccessible and difficult places to work [1].

The literature on the mobility of health workers suggests that an interplay of “push” and “pull” factors influence an individual’s decision to leave or stay in a rural workplace. Pull factors can attract health workers to urban workplaces or even abroad. These include career advancement, such as positions in centres of medical and educational excellence, higher financial rewards and improved living conditions. Concurrently, push factors, such as professional isolation, poorly resourced facilities and limited recreational possibilities, can provoke health workers to leave [2].

In general, retention is known to be influenced by personal origin, family and community factors, financial considerations, career development, working and living conditions, as well as mandatory service requirements [1,3]. Rural upbringing of physicians has been associated with a willingness to engage in rural practice [4]. Moreover, spouses with a rural upbringing have also been found to integrate more easily with rural communities [5].

The Philippines suffer from a maldistribution of health workforce, with only 10% of doctors, dentists, and pharmacists found in rural areas where more than half of the population resides [6]. In 1993 the ‘Doctors to the Barrios’ (DTTB) Program was launched. At the outset the vision was that all municipalities in the Philippines would have a doctor within 20 years. The program was established in the wake of the devolution of health services administration to local government level in 1991. The Local Government Code that formalised the process granted local government units (LGUs) administrative autonomy which allowed them to allocate budgets for health services as they saw fit [7]. Devolution effectively transferred the management of health workers to local politicians with little or no experience in managing health systems. The administrative transition thus led to an initial decline in morale of health staff and resignation of key personnel [7]. Despite the financial autonomy of the LGU, rural LGUs found it difficult to offer incentives that attracted health personnel as compared to urban areas [8]. By 1992, a rapid national survey identified 271 municipalities to be without doctors and the Department of Health (DOH) launched the DTTB Program as a response to this shortage.

The DTTB Program regularly assigns a cohort of physicians to underserved and difficult-to-access municipalities for a period of 2 years. Initially, the deployment was twice or three times a year. During this assignment, the physicians receive good salaries and full benefits as employees of the national DOH. Later on, they have priority access to a Master’s degree or to clinical residency programs. These physicians have the role of Municipal Health Officer (MHO) which includes technical and managerial functions that cover national and local policy and program implementation, financial effectiveness, human resource for health management and development, provision of health services, information management, and infrastructure development and preservation. After completion of the two years, the DTTBs have the choice to remain in their position and to be “absorbed” as MHO of the LGU. However, LGU employees in low-income (5th and 6th class) municipalities are only entitled to part (65-70%) of what they would normally receive from a national agency under the same salary grade. Consequently, DTTBs experience a drop in their total compensation upon absorption.

The DOH eventually expanded the deployment criteria to take into account population growth such that more than one doctor is required to meet the needs of the municipality, and to temporarily replace MHOs on study leave. A scheme of biannual Continuing Medical Education (CME) as a further incentive for participants was also introduced. By 2005 the Philippines were experiencing alarming levels of out-migration of doctors. The media contrasted the image of doctors in high paying jobs abroad with that of altruistic doctors choosing to serve in rural villages across the Philippines. This heroic portrayal of DTTBs increased attention for the Program leading to higher application rates and greater political backing. In the light of these developments, public medical schools began to make the receipt of scholarships dependent upon a period of mandatory service after qualification as a physician. The current cohort of DTTBs is the second batch that can make their CME sessions count towards a Master’s degree in Public Management with a major in Health Systems and Development offered through a partnership between the DOH and the Development Academy of the Philippines.

**Figure 1:**
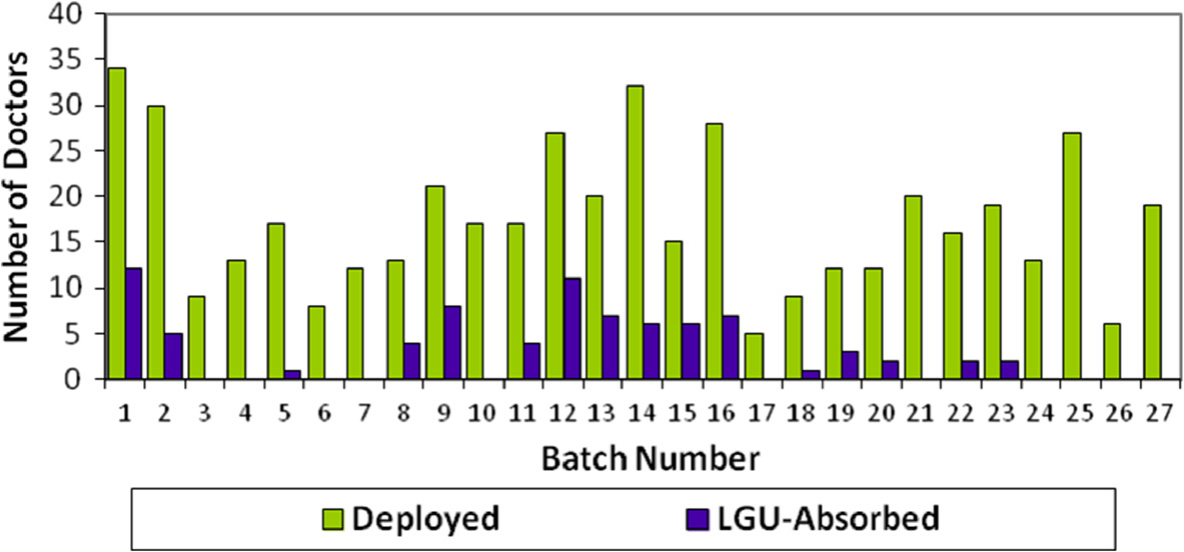
**Number of physicians deployed under the Doctors to the Barrios (DTTB) Program and absorbed by the Local Government Unit (LGU).** The first batch was deployed in1993 and batch 27 in 2009.

Ongoing monitoring by the DOH showed that of the 452 DTTBs who took part in the Program between 1993 and 2011, only 81 (18%) chose to remain in their rural posts and to be absorbed by their respective LGUs. According to the DTTB Alumni Database, the numbers of those choosing to be absorbed have in fact declined since 2006 (Figure 1).

Against this backdrop, in April 2011 the DOH requested an assessment of the DTTB Program to find out why only so few physicians choose to remain in their rural post after the initial 2 years have elapsed. The assessment was guided by the questions: why do physicians join the DTTB Program, what factors influence their decision to remain or leave their rural posts, what could the Program do to better respond to the needs of the physicians whom it deploys and to increase retention. The results of this assessment are presented here.

## Methods

For the assessment of the DTTB Program the researchers had the opportunity to meet with all the current DTTBs (n=71) on the occasion of a forthcoming national CME session, and to a DTTB alumni database of the 452 former Program participants – although, as explained below, the contact information was later found to be outdated which severely compromised its usefulness for random sampling and tracing purposes. Given this constellation, the changes in the design of the Program that had taken place during the years of its existence and constraints of time and other resources, a mixed methods approach was selected: a self-administered questionnaire for all current DTTB, and oral interviews by telephone with available former DTTBs. The findings from both methods were analysed separately and later consolidated for the final interpretation [9].

The development of the questionnaire for current DTTBs was based on a review of formally published as well as grey literature on staff retention in rural areas. A review of policy documents from the Philippines and the DTTB Program was also conducted. These included the DOH Revised Operational Guidelines for the Implementation of the DTTB Program and the Memorandum of Agreement between the DTTB, the LGU, and the DOH. Moreover, explorative interviews were conducted with key informants including the Director of the DOH Health Human Resource Development Bureau, and the past and present DTTB Program coordinators in order to guide the development of the questionnaire.

The questionnaire used in this study was inspired by The ‘Stayers Questionnaire’ used in a health workforce assessment manual in Uganda [10]. Permission was kindly given by the author, Emily Bancroft of the University of Washington, to adapt this tool for the purpose of the assessment. The tool covers the constructs: personal and job satisfaction, career advancement, working environment, living conditions, and compensation. These were all issues that the key informants had raised as being relevant for the assessment. In addition constructs dealing with local politics and DOH support were added. Each of the constructs comprises a list of statements. The respondents were asked to use a 5-point Likert scale to indicate whether they strongly agreed, agreed, were neutral, disagreed or strongly disagreed with the statements. 41 of the 77 statements from the original ‘Stayers Questionnaire’ were used in the version employed for this assessment.

The variables retained and explored from the demographics section of the original Stayers Questionnaire were sex, marital status, number of dependents, and city/municipality of residence. After perusing the responses from the key informant interviews it was decided to expand these variables to include whether respondents graduated from a medical school within or outside the National Capital Region (NCR), whether their main reason to join the Program was to return service for a scholarship or not, whether their main reason for joining was an interest in Public Health/Community Medicine or other reasons, and whether they planned to remain in the area of assignment for more than one year or indefinitely after the Program, or alternatively to leave.

To reach the current DTTBs in an efficient manner, advantage was taken of a CME session to request their participation in the assessment. All of the current 71 DTTBs were in attendance and all agreed to take part. Time was allocated at the session to introduce the study and the use of the Likert scale. Completing the questionnaire took approximately 30 minutes. In all but one case, the participants responded to all items of the questionnaire.

Epi Info was used to analyse data obtained by the questionnaires from current DTTBs. The descriptive aspect of the analysis measured the frequencies by which the respondents gave a rating of “agree strongly” or “agree” to a specific question and the corresponding mean score. For the inferential component of the analysis, independent two-sample *t*-tests were run for the statements and questions, and the differences between the mean scores were tested for statistical significance (p-value ≤ 0.05).

The topic guideline for interviews with former DTTBs was developed to broadly align with the sections of the self-administered questionnaire and made use of open questions so as to obtain further, clarifying and explanatory information. Both tools were tested by a small panel including two former DTTBs, a former DTTB Program Coordinator and the researchers conducting the assessment. The topic guideline provided the key themes for the analysis of the interviews with former DTTBs. Sub-themes emerging from the responses were then identified.

Tracing the former DTTBs proved to be extremely difficult, as the contact information in the Alumni Database had not been maintained. Snowball sampling was, therefore, used over a one-month period to generate a productive sample [11], that included DTTBs who chose to remain in their rural position after the Program, some who left, and others who remained in rural service, but preferred to re-enter the Program in a different geographical area. An effort was also made to include former DTTBs from different cohorts since the Program’s inception.

Former DTTBs (n=26) were interviewed either face-to-face or, in the majority of cases for practical reasons, by phone. All the interviews were conducted by the same interviewer (JAL). None of those approached refused to be interviewed. The interviews lasted an average of 20 minutes. The responses were translated into English and directly transcribed by the interviewer.

This assessment was suggested and facilitated by the Human Resources Development Bureau which obtained ethical clearance from the Department of Health in the Philippines. Informed consent was gained from all those who agreed to take part in either the questionnaire or the interviews. Respondents were allowed to withdraw at any time or skip questions without having to give a reason. However, no one made use of this possibility. All data were de-identified during the analysis so that responses could not be traced back to a particular informant.

## Results

Of the 71 current DTTBs who filled out the self-administered questionnaire, 46 (65%) were female and 25 (35%) male. The mean age of the respondents was 29 years. Twenty-nine (41%) of the respondents had dependents, with an average of 2 dependents each. Eleven (15%) of them were married. Fifty-eight (82.9%) were from urban areas and 45 (63.4%) graduated from medical schools in the NCR.

Of the 452 DTTBs who had graduated from the Program, a total of 26 DTTBs took part in the interviews; 14 of whom were male and 12 were female. The mean age of the interviewees was 38 years, with a range of 28 to 64 years. Fifteen of them came from a rural background. Nine of the interviewees were employed as local government health officers, 7 worked under the DOH Central Office, 5 were hospital clinicians, 3 were private practitioners, and 2 worked in other public health agencies. Twelve of the interviewees had chosen to work as municipal health officers for an average of 6 years after completing the DTTB Program, while 6 had re-entered the Program for a further rural deployment but to a different municipality. The remaining 8 had left the rural assignment after finishing the Program.

### Personal satisfaction

The perception of current DTTBs with respect to their work situation is detailed in Table 1. Personal satisfaction was high and most current DTTBs felt respected, fulfilled and appreciated by their communities, supported by their families and had friends at work. Although there is less agreement with the statements about appreciation from the municipal government and their primary employer (DOH), there is still a general sentiment of appreciation.

**Table 1:** Personal satisfaction of current DTTBs (n = 71)

**To what extent do you agree with the following statements? (*5=strongly agree, 4=agree, 3=neutral, 2=disagree, 1=strongly disagree)***	**Number who agree or strongly agree**	**Mean (Std. Dev.)**
My opinion matters at work; I feel respected.	66 (92.9%)	**4.32** (0.65)
The community in general to which I am assigned appreciates my work.	63 (88.7%)	**4.24** (0.64)
My family supports my decision to work as a DTTB.	62 (87.3%)	**4.35** (0.79)
I find fulfilment in serving my community.	60 (84.5%)	**4.38** (0.78)
Considering everything, I am satisfied with my job.	60 (84.5%)	**4.08** (0.63)
I have a good friend(s) at work.	59 (83.1%)	**4.17** (0.74)
The municipal government to which I am assigned appreciates my work.	56 (78.8%)	**4.00** (0.70)
The ***DOH*** appreciates my work.	53 (74.6%)	**3.92** (0.73)

Former DTTBs derived personal satisfaction from getting to know a new part of the country with different cultures, meeting new people and being accepted in a rural community.

"“[What I find to be most satisfying is] experiencing new cultures and learning to live with them, while at the same time making friends from the DTTB Program.” (Female DTTB, deployed 2004)"

### Job satisfaction and career development

Most of the current DTTBs found their work meaningful and stimulating as shown in Table 2. The majority were clear about what is expected from them and considered they have opportunities for career development. Fewer agreed that they were provided with adequate prior training for their role. The statements pertaining to the fairness of evaluation and support from municipal government and the DOH Central Office drew only neutral responses. The majority of current DTTB were not satisfied with the quality of care that their health centres provide.

**Table 2:** Job satisfaction and career development of current DTTBs (n = 71)

**To what extent do you agree with the following statements? (*5=strongly agree, 4=agree, 3=neutral, 2=disagree, 1=strongly disagree)***	**Number who agree or strongly agree**	**Mean (Std. Dev.)**
I enjoy working as a DTTB; the work I am doing is meaningful and stimulating.	64 (90.1%)	**4.23** (0.61)
When I come to work, I know what is expected of me.	57 (80.3%)	**3.92** (0.73)
I feel that there are sufficient opportunities to develop career-wise.	52 (73.2%)	**3.80** (0.84)
The job matches my skills and experience.	50 (70.4%)	**3.85** (0.77)
I receive recognition for doing good work.	48 (67.6%)	**3.69** (0.84)
I am satisfied with the support I receive from the DOH Regional Office.	44 (62.0%)	**3.61** (0.92)
I receive encouragement to develop myself from DOH staff or LGU officials	43 (60.6%)	**3.66** (0.83)
I have been given the training needed to perform the work expected of me.	43 (60.6%)	**3.55** (0.94)
I am fairly evaluated on my work.	41 (57.7%)	**3.56** (0.63)
I am satisfied with the support I receive from the *municipal government*.	41 (57.7%)	**3.46** (0.89)
I am satisfied with the support I receive from the *DOH Central Office*.	40 (56.4%)	**3.55** (0.89)
I am satisfied with the quality of care that my health center can provide.	26 (36.6%)	**3.10** (0.97)

Former DTTBs derived job satisfaction from applying their training in practice, gaining experience, successfully lobbying for staff benefits and development of their respective health centres, as well as from improvement in local health indicators. Some former DTTBs perceived a decline in DOH support once they were absorbed by their LGU. More than half of them enjoyed opportunities for further education while being on the Program. However, others mentioned that training on legal issues was lacking. Others were dissatisfied with their administrative roles as MHO, had concerns that their clinical skills could become out-dated, or had left to undergo further training in clinical areas.

"“I want to have more career options so that after [clinical] residency I can choose between private practice or public health.” (Female DTTB, deployed 2008)"

### Work environment

Current DTTBs considered their workloads to be manageable and that they had the flexibility to attain a reasonable work-life balance as shown in (Table 3). Staff morale was sufficient to create a pleasant work environment and utilities were generally available in the health facilities. Less than half of these DTTBs agreed that there was good access to essential drugs and resources for health programs. Most disagreed about the availability of other medical supplies and equipment. Moreover, less than half perceived their LGU to be competent.

**Table 3:** Work environment of current DTTBs (n = 71)

**To what extent do you agree with the following statements? (*5=strongly agree, 4=agree, 3=neutral, 2=disagree, 1=strongly disagree)***	**Number who agree or strongly agree**	**Mean (Std. Dev.)**
I can take time to eat lunch and snacks every day.	58 (81.7%)	**4.06** (0.81)
The workload is manageable.	56 (78.9%)	**3.83** (0.72)
I have flexibility to balance the demands of my workplace and my personal life.	54 (76.1%)	**3.83** (0.68)
I have regular electricity at my workplace.	52 (73.3%)	**3.83** (1.12)
I have a pleasant work environment; I am satisfied with the morale level of my health center staff.	49 (69.0%)	**3.66** (0.84)
I have access to clean running water at my workplace.	44 (62.0%)	**3.42** (1.21)
My health center has good access to essential drugs and medications.	30 (42.2%)	**3.07** (1.09)
I work with a competent LGU	28 (39.4%)	**3.25** (0.82)
My Rural Health Unit has access to resources for health programs and projects.	21 (29.5%)	**3.11** (0.90)
I have the supplies which I need to do my job well and safely.	19 (26.8%)	**2.75** (1.01)
I have the equipment which I need to do my job well and efficiently.	13 (18.3%)	**2.61** (0.98)

The former DTTBs described the difficulties of their role brought about by weaknesses in local governance, perceived inadequacy of LGU support, and local politics.

"“It is hard to work with many bosses – you have to satisfy many politicians who sometimes have conflicting political interests.” (Male DTTB, deployed 1999)."

Relations with the municipality mayor, as the MHO’s direct superior, emerged as extremely important in this regard. Former DTTBs who re-entered the Program rather than choosing to be absorbed felt that, as part of the LGU, it would be harder to critique the system and create positive change. Former DTTBs who stayed after the 2 years all stated that support from their respective LGUs was crucial to their decision to stay.

### Living and community conditions

Current DTTBs reported having comfortable accommodation with a clean toilet, regular electricity and, to a lesser extent, running water as shown in (Table 4). Most considered themselves part of their community and reported feeling safe. Levels of agreement are lower when it comes to transportation and availability of supplies for personal needs. Only a quarter of the respondents agreed that there were adequate options for leisure and entertainment.

**Table 4:** Living and community conditions of current DTTBs (n = 71)

**To what extent do you agree with the following statements? (*5=strongly agree, 4=agree, 3=neutral, 2=disagree, 1=strongly disagree)***	**Number who agree or strongly agree**	**Mean (Std. Dev.)**
My accommodation has a comfortable place to sleep.	58 (81.7%)	**4.11** (0.77)
My accommodation has a clean toilet and shower.	58 (81.7%)	**4.10** (0.72)
I consider myself a part of the community to which I am assigned.	53 (74.6%)	**3.90** (0.80)
I have regular electricity at my accommodation.	52 (73.2%)	**3.75** (1.07)
I feel safe in my area of assignment.	49 (69.0%)	**3.76** (0.82)
I have access to clean running water at my accommodation.	47 (66.2%)	**3.75** (0.97)
I have safe and efficient transportation to work.	42 (59.2%)	**3.55** (1.00)
Supplies for my personal needs are available in my area of assignment.	39 (55.0%)	**3.54** (0.88)
My area of assignment has sufficient options for leisure and entertainment.	18 (25.3%)	**2.73** (1.11)

Many of the former DTTBs agreed that problems with living conditions influenced their decision to leave their rural post and those who stayed on reported having good accommodation. Negative issues raised in the interviews included difficulties with reaching and living in storm-prone regions and political violence during election times.

"“I did not feel very safe in my area of assignment. Elections were approaching and there were reports of politically-motivated killings.” (Female DTTB, deployed 2007)"

Physical separation from one’s family and relatives was frequently mentioned as a push factor. However, some indicated that with the improved means of communication offered by internet and mobile phones the situation today may be less difficult.

### Compensation

Most current DTTBs considered their salary and benefits to be fair. Less agreed regarding the representation and travel allowances as shown in (Table 5).

**Table 5:** Salary, benefits, and incentives of current DTTBs (n = 71)

**To what extent do you agree with the following statements? (*5=strongly agree, 4=agree, 3=neutral, 2=disagree, 1=strongly disagree)***	**Number who agree or strongly agree**	**Mean (Std. Dev.)**
My salary is fair	56 (78.9%)	**3.97** (0.83)
My benefit package is fair	51 (71.9%)	**3.80** (1.04)
My representation and travel allowances are fair	40 (56.3%)	**3.49** (1.08)

Former DTTBs considered conditions to have been good but felt better compensation was justified, as they were practically on-call for 24 hours and carried a great deal of responsibility. Some of the former DTTB who were absorbed, described having received inadequate allowances and incentives from the LGU and how they engaged in private enterprise activities as a response. As such possibilities were limited in rural areas, the constrained rural economy ultimately became a reason to leave.

### Main reasons for joining the program

Table 6 shows that more than half the current DTTBs acknowledged that mandatory rural service as part of their medical scholarship was their main reason for joining the DTTB Program (referred to hereafter as “return service*”*). Almost a quarter entered the Program to help those living in rural areas, while several respondents cited their interest in public health and community medicine.

**Table 6:** Main reasons for joining the DTTB Program (n = 71)

**What is/are your main reason(s) for joining the DTTB Program? *Respondents may give more than one answer***	**Number of respondents who gave this answer**
Return Service	38 (53.5%)
Opportunity to Serve	17 (23.9%)
Interest in Public Health and Community Medicine	13 (18.3%)
Experience and Adventure	6 (8.5%)
Fulfilment and meaning in life	3 (4.2%)
Master’s degree and career opportunities	2 (2.8%)
Good salary	1 (1.4%)

By contrast, former DTTBs attached most importance to having the opportunity to help rural populations. Many described how their decision to join the Program was influenced by community exposure and encounters with rural physician role-models during their medical studies and rotations in government hospitals. Several mentioned joining the Program for adventure and travel, although only one with this motivation remained in service after the two years. Former DTTBs make no mention of return service. This was to be expected, as this reflects a more recent trend of conditionality that has been subsequently introduced by public medical schools and scholarship foundations.

### Associations between variables

Associations between variables were next analysed from the data collected from the current DTTBs. Only those that were found to be statistically significant are considered in the following:

Differences between sexes (Table 7) revealed that male DTTBs felt significantly more respected and were more likely to have good friends at work than females.

**Table 7:** Differences between sexes

**To what extent do you agree with the following statements? (*5=strongly agree, 4=agree, 3=neutral, 2=disagree, 1=strongly disagree)***	**Mean (Std. Dev.)**		**p-value**
	**Male n=25**	**Female n=46**	
My opinion matters at work; I feel respected.	**4.56** (0.51)	**4.20** (0.69)	+
I have a good friend(s) at work.	**4.44** (0.58)	**4.02** (0.77)	+

p-value legend: + = p ≤ 0.05.

DTTBs who joined the Program because of return service enjoyed significantly lower personal satisfaction than those who joined the Program for other reasons (Table 8). Other less positive perceptions were also linked with mandatory service obligations.

**Table 8:** Main reason for joining is return service for scholarship

**To what extent do you agree with the following statements? (*5=strongly agree, 4=agree, 3=neutral, 2=disagree, 1=strongly disagree)***	**Mean (Std. Dev.)**		**p-value**
	**Return Service n=38**	**Other Reasons n=33**	
Considering everything, I am satisfied with my job.	**3.84** (0.44)	**4.36** (0.70)	+++
I have a good friend(s) at work.	**3.95** (0.70)	**4.42** (0.71)	++
I find fulfilment in serving my community.	**4.08** (0.78)	**4.73** (0.63)	+++
The job matches my skills and experience.	**3.63** (0.67)	**4.09** (0.80)	++
I enjoy working as a DTTB; the work I am doing is meaningful and stimulating.	**3.92** (0.54)	**4.58** (0.50)	+++
I feel that there are sufficient opportunities to develop career-wise.	**3.58** (0.89)	**4.06** (0.70)	++
I have a pleasant work environment; I am satisfied with the morale level of my health center staff.	**3.39** (0.82)	**3.97** (0.77)	+++
The workload is manageable.	**3.66** (0.81)	**4.03** (0.53)	+

p-value legend: + = p ≤ 0.05; ++ = p ≤ 0.02; +++ = p ≤ 0.005.

By contrast, DTTBs who joined the Program because of their interest in public health or community medicine were significantly more likely to find fulfilment in their work with rural communities (Table 9).

**Table 9:** Main reason for joining is interest in Public Health or Community Medicine

**To what extent do you agree with the following statements? (*5=strongly agree, 4=agree, 3=neutral, 2=disagree, 1=strongly disagree)***	**Mean (Std. Dev.)**		**p-value**
	**Public Health n=13**	**Other Reasons n=58**	
I find fulfilment in serving my community.	**4.85** (0.38)	**4.28** (0.81)	++

(++ = p ≤ 0.02).

Two thirds of the DTTBs who graduated from a medical school in the NCR joined the Program to return service. To avoid confounding, the participants whose main reason for joining the Program was return service were excluded from these *t-*tests.

Graduates of medical schools from the NCR were less satisfied with DOH support compared to those who graduated from schools in the provinces (Table 10). The graduates from the NCR also found less flexibility to enjoy their personal time and were less likely to find sufficient options for leisure and entertainment. They were more likely to disagree with the statement that health programs are sufficiently resourced and significantly less satisfied with their compensation.

**Table 10:** Graduated from a medical school in the National Capital Region (NCR)*

**To what extent do you agree with the following statements? (*5=strongly agree, 4=agree, 3=neutral, 2=disagree, 1=strongly disagree)***	**Mean (Std. Dev.)**		**p-value**
	**NCR n=15**	**Non-NCR n=18**	
I am satisfied with the support I receive from the DOH Central Office.	**3.07** (1.03)	**3.89** (0.68)	++
I am satisfied with the support I receive from the DOH Regional Office.	**3.20** (1.01)	**3.94** (0.73)	++
I have flexibility to balance the demands of my workplace and my personal life.	**3.67** (0.49)	**4.22** (0.55)	+++
My Rural Health Unit has access to resources for health programs and projects.	**2.67** (0.82)	**3.61** (0.78)	+++
My area of assignment has sufficient options for leisure and entertainment.	**2.07** (1.16)	**2.89** (0.90)	+
My salary is fair.	**3.60** (0.83)	**4.33** (0.59)	++
My representation and travel allowances are fair.	**2.67** (0.90)	**3.89** (1.13)	+++

p-value legend: + = p ≤ 0.05; ++ = p ≤ 0.02; +++ = p ≤ 0.005.

*Excluding respondents with return service obligations.

Only 7 of the 71 current DTTBs declared plans to remain in their rural post for more than one year or indefinitely after completing the Program. The mean age of these physicians was 28 years, 4 of them were male, 3 were married, 4 had dependents, 5 originated from urban areas, 4 trained in the NCR and 3 had entered the Program to return service.

DTTBs who planned to remain in the Program gave significantly higher ratings to the support they received from the DOH Regional Office and the municipal government (Table 11). They were also more likely to agree that their health center had good access to essential drugs and that they worked for a competent LGU. Furthermore, those who planned to remain found adequate options for leisure and entertainment in their respective areas of assignment.

**Table 11:** Differences between those who plan to stay or leave

**To what extent do you agree with the following statements? (*5=strongly agree, 4=agree, 3=neutral, 2=disagree, 1=strongly disagree)***	**Mean (Std. Dev.)**		**p-value**
	**Stay n=7**	**Leave n=64**	
I am satisfied with the support I receive from the DOH Regional Office.	**4.29** (0.76)	**3.53** (0.91)	+
I am satisfied with the support I receive from the municipal government.	**4.14** (0.69)	**3.39** (0.88)	+
My health center has good access to essential drugs and medications.	**3.86** (0.90)	**2.98** (1.08)	+
I work with a competent LGU	**4.00** (0.82)	**3.17** (0.79)	++
My area of assignment has sufficient options for leisure and entertainment.	**3.71** (0.76)	**2.63** (1.09)	++

p-value legend: + = p ≤ 0.05; ++ = p ≤ 0.02; +++ = p ≤ 0.005.

### Most important deciding factors for retention

LGU support was the most frequently cited factor followed by compensation, factors related to family concerns and career advancement opportunities as shown in (Table 12). Former DTTBs who remained in their areas of assignment indicated that both intrinsic and extrinsic factors played a role, with mention made of the importance of responding to a felt need, making a difference, enjoying the work, and affinity with the community. Extrinsic influences included marriage to a local resident, offers of higher education, or whether the LGU provided adequate benefits.

**Table 12:** Most important deciding factors for retention (n = 71)

**What would be the most important deciding factors for you to remain in your area of assignment after your term as a DTTB? *Respondents may give more than one answer***	**Number of respondents who gave this answer**
Local government support	31
Good salary and compensation	20
Family (distance from family, finding a spouse in the community, livelihood opportunities for spouse)	10
Career advancement opportunities	8
Sustainability of health projects	6
DOH support and re-centralized health human resources	5
Needs of the poor and underserved	3
Ease of transportation	1
Passion for public health	1
Personal reasons	1

However, most of the former DTTB who initially remained in their rural post after the Program eventually left. The most frequently cited reasons for this were related to family and career development. Specific family reasons included the need to spend more time with their children, to raise their children in the city or at least in their hometowns, and to be within reach of their aging parents or relatives as the doctor in the family.

"“My father died and I was not there [for him]. I don’t want this to happen to my mother.” (Male DTTB, deployed 2002)"

Those who did not continue in their rural post after completing the Program also stated career advancement as a major reason for leaving. Several preferred to undertake further clinical training to have more flexibility in their choice of work in the future. Some left because they felt they had done their part for the underserved, or from the frustration of witnessing the poverty in the rural areas.

### Suggestions to improve retention

Better compensation was the most frequent suggestion made by both former and current DTTBs to encourage remaining in their rural posts. The under-provision of prescribed benefits and the previously described reduction in total compensation upon absorption by the LGU were shown to be key issues. Increased remote-assignment allowances and benefits, including support for dependents, were suggested as ways to mitigate this situation.

Former DTTBs who chose to remain in their posts highlighted how the DTTB Program itself was instrumental in their decision by having enabled them to face the challenges of their role or facilitated a smooth transfer to the LGU. In this regard, the DOH, as the implementer of the DTTB Program, was considered to play a crucial role in lobbying for LGU support. In some cases, the experience derived during the 2 years in the Program drove the desire to serve and was also instrumental in the decision to stay. Former DTTBs who decided not to remain in their posts, nonetheless attributed to the Program their interest in pursuing a career in public health.

Lack of LGU and DOH support were frequently mentioned as impediments to retention. However, no concrete measures to improve LGU support were proposed by the DTTBs that took part in this study. As for the DOH, it was proposed that they improve the technical, operational, career development, legal, and even moral support (e.g. improving communication and interaction with the regional office) provided to DTTBs. Further suggestions included the targeted recruitment of physicians with rural backgrounds, particularly those who are from the areas included in the Program, tailored career-development coaching for those who choose to stay in the rural areas, improved pre-deployment orientation to better prepare the DTTBs for their roles and the introduction of a rural physician network.

## Discussion

### Effect of reasons for joining the program

Job satisfaction among health workers has been found to positively correlate with greater work commitment and retention [12,13]. While many of the former DTTBs reported being satisfied because their role allowed them to make a difference, the situation regarding the satisfaction of current DTTB is more complex. Those who joined to return service experienced less job satisfaction. In particular, graduates from the relatively affluent NCR complained about their compensation and leisure possibilities. It can thus be implied that these respondents are less likely to remain in rural service.

On the other hand, those who joined out of an interest in public health were more satisfied with their rural work. It is arguable that the current DTTBs have a stronger focus upon themselves and their individual needs and wishes. This might be the effect of the changing profile of Program applicants over time, or actually reflect a shift in societal values.

### Factors influencing DTTBs in their decision to leave or remain

The LGU was shown to play a pivotal role in retention and has the most important influence on DTTBs’ decision-making. It becomes apparent that, if LGU support is in place, then the MHO can rely on the availability of utilities, infrastructure, logistics, services, funding, and human resources – thus considerably facilitating the management of the local health system. Conversely, where LGU support is lacking, personal security, the availability of drugs, medical supplies, health program funds and even the provision of DTTB benefits become problematic. Moreover, once DTTBs are absorbed by the LGUs, they become more susceptible to the pressures of local politics. This would explain why some former DTTBs preferred to re-enter the Program and be assigned to another area and to retain their more neutral DOH employee status than to become an LGU employee.

DOH support is also important for retention, particularly when DTTBs consider or embark upon absorption. Some of the former DTTBs expressed regret that DOH support waned once they became part of the LGU despite efforts from the DOH to continually contact and invite former DTTBs to CME sessions. This implies that former DTTBs may have either been poorly informed of the support available to them or they may have expected support in other areas, particularly in dealing with local governance issues. DTTBs graduating from medical schools in NCR were significantly less positive about DOH support. This could be because these physicians are overall less likely to remain in their rural post and perceive the linkage with the DOH to be crucial for their immediate and future careers.

Career advancement is one of the most common reasons why former DTTBs gave up being a rural physician. Many of those who joined the Program were doctors at the start of their careers and still quite mobile career-wise. Meanwhile, DTTBs who choose to be absorbed face less opportunities of going up the career ladder, especially because the position of MHO is typically the highest non-elected position for a doctor in the municipal government. The lack of career mobility is an established problem in decentralized settings. Aside from the difficulties involved in moving up to the national level and between devolved administrative units, information regarding career opportunities is hardly shared [14].

The majority of the current cohort are females, yet female DTTBs are significantly less likely to feel respected and have good friends at work than their male counterparts. This may indicate that women find it more difficult to deal with local politics in rural areas. It is also likely that men dominate the key LGU positions in such areas.

Most current DTTBs consider their workloads to be manageable and agree that utilities such as water and electricity are generally available at their respective health facilities. However there is strong disagreement with the statements regarding the availability of medical supplies and equipment. DTTB who intend to remain in their rural posts are significantly more likely to be satisfied with the availability of essential drugs. It is worrying that the majority of current DTTB are found to be dissatisfied with the quality of care that their health centres provide. Whilst not specifically articulated, it is possible that this reflects a frustration with the possibilities of rural practice that may erode DTTB readiness to remain in their post.

Most current DTTB have adequate accommodation and feel safe in their communities. The interviews with former DTTBs revealed that insecurity, due to natural hazards or political tensions, can rapidly undermine a physician′s readiness to remain, as documented in other studies in the Philippines [15].

Family issues also considerably influence retention. The DTTBs mentioned the need to be closer to aged parents as a greater constraint to retention than the needs of spouses and children, which are more commonly cited in the literature [16,17]. It is possible that the importance of parental support for DTTBs, perhaps in admiring their idealism or being fearful for their safety, may have been underestimated. Moreover, family expectations may put increased pressure on physicians, as they get older, to maintain a standard of living not found in rural areas [18]. Compensation plays a prominent role in the decision-making of DTTBs. While most current DTTBs agree that they are fairly compensated, the views of former DTTBs show that this is not the case after absorption. The issue of the effective salary downgrade has haunted the DTTB Program since its conception. The suggestions made by respondents to offset this with other benefits and allowances need to be examined by the Program management. However, the findings suggest that increasing compensation as a stand-alone measure will not be sufficient [19], but needs to go hand in hand with efforts to strengthen LGU capacity and support.

### How the DTTB Program can increase retention

The findings suggest that the DTTB Program could gain from exploring physicians’ backgrounds and reasons for joining. There could be potential to more actively recruit physicians from rural areas, or to consider strategies, including advocacy and scholarships, to encourage rural students to consider medicine as a future area of study. Reasons for joining could be examined during interviews or through letters of motivation. Priority should be given to qualified physicians with an interest in public health and community medicine and who trained outside of NCR. In particular, a decision should be taken whether to continue to automatically accept return-service physicians. Given that these physicians have already enjoyed the benefit of a scholarship based on an agreement to undertake rural service, it can be argued that they can be directly assigned to rural municipalities as part of the terms of their contract, thus foregoing the issues related to the transition of employment from the DOH to the LGU. For physicians with no obligation to return service, the DOH could consider involving LGUs in the choice of DTTBs from the outset to maximise the chances of a harmonious relationship between the two parties. The experience of the University of the Philippines Manila School of Health Sciences which promotes close early contact, and the elaboration of a social contract, between health workers and partner LGUs could be drawn upon here [20].

On the other hand, it should be noted that 3 of the 7 physicians who planned to extend beyond the initial 2 years had originally joined the Program to return service. The number of those planning to continue is low and their profiles are so disparate that generalisations cannot be made. Yet, every physician counts in such a context as observed in the rural Zamboanga province of the Philippines, where the presence of just a small number of physicians already had a positive effect on community health outcomes [15]. A network of rural physicians could be usefully established to facilitate communication and experience sharing and to advocate for greater political support for rural health issues.

The role of MHO involves both administrative and clinical skills*.* For DTTBs who intend to work as clinical specialists in the future, the DOH needs to offer more opportunities for clinical updates and rotations in centres of medical excellence. For those that see their future in more managerial roles, the currently offered Masters course in Public Management appears to be a good choice.

## Conclusion

This assessment suggests that the DTTB Program needs to work on enhancing the factors that drive retention, whilst mitigating those that impede it. Through improved collaboration with the DOH, LGUs need to strengthen the support provided to DTTBs. The findings suggest 5 areas for future attention:

The motivation to join the DTTB Program was, in some cases, awakened through meetings with inspiring rural physicians and rotations undertaken during training. This underlines the importance of continued advocacy at medical schools, with a particular focus on those located outside of the NCR, and of ensuring that all medical students are routinely afforded exposure to rural practice.

DTTB Program coordinators would be well advised to invest in exploring why individuals seek to join. Priority should be given to physicians motivated by a wish to help rural populations or by an interest in public health, to those who have trained outside of NCR and those originating from rural or disadvantaged areas. Whether physicians should be able to use the Program to fulfil return service obligations should be critically assessed.

The DTTB preparation provided by the DOH needs to be intensified with regards to political, legal and administrative aspects and made more gender-sensitive. Modern communication technology should be made available in MHO offices.

Greater efforts are needed to ensure that absorbed DTTBs continue to receive DOH support so they can keep abreast with new clinical developments, and access new positions readily once the agreed retention period has been completed.

Finally, most DTTBs will not remain in a rural area for an open-ended period of time. The retention period that is aspired to needs to be more clearly defined and aligned with LGU timetables, career development options and succession planning. The assessment highlights the importance for the DTTB Program to invest in an effective monitoring and tracking system.

## Abbreviations

CME: Continuing Medical Education; DOH: Department of Health; DTTB: Doctor to the Barrios; LGU: Local Government Unit; MHO: Municipal Health Officer; NCR: National Capital Region.

## Competing interests

The authors all declare that they have no competing interests.

## Authors’ contributions

JAL conceived the study, undertook the literature review, adapted the questionnaire, developed the interview guideline, collected the data, conducted the analysis, and wrote the first draft of the manuscript and took the lead for all later versions. HP assisted with the literature review, with data analysis, contributed at all stages of manuscript preparation including submission. KR contributed to the study design, provided clearance for the study, supervised the thesis, coordinated the data collection, and contributed to the background and discussion. RN contributed to the study design, tested the tools, coordinated the data collection, and contributed to the background and discussion. AR contributed to the study design, oversaw development of the tools, supervised the thesis, revised the later stages and final version of the manuscript. All authors read and approved the final manuscript.

## Pre-publication history

The pre-publication history for this paper can be accessed here:

http://www.biomedcentral.com/1472-6963/12/411/prepub
